# Parental Illness and Life Satisfaction among Young People: A Cross-Sectional Study of the Importance of School Factors

**DOI:** 10.3390/ijerph19052719

**Published:** 2022-02-26

**Authors:** Sanne Ellegård Jørgensen, Lau Caspar Thygesen, Anette Andersen, Pernille Due, Susan Ishøy Michelsen

**Affiliations:** 1 National Institute of Public Health, University of Southern Denmark, Studiestræde 6, 1455 Copenhagen, Denmark; lct@sdu.dk (L.C.T.); pdu@sdu.dk (P.D.); simi@sdu.dk (S.I.M.); 2 Steno Diabetes Center Aarhus, Aarhus University Hospital, Palle Juul-Jensens Boulevard 99, 8200 Aarhus, Denmark; anette.andersen@rm.dk

**Keywords:** parental illness, adolescents, school environment, life satisfaction

## Abstract

Background: The aim of this study was to examine the relationship between parental illness and life satisfaction among Danish adolescents and the potential modifying effect of positive school experiences. Moreover, we describe the use of student counsellor services among adolescents with and without ill parents. Methods: Data included 9565 adolescents primarily aged 13–19 years, who participated in the cross-sectional Well-being Despite Study. Multilevel logistic regression models including joint effect analyses were performed. Results: Parental illness was strongly associated with life satisfaction. Negative school experiences were more frequent among adolescents with ill parents and strongly associated with low life satisfaction for all students. However, joint effect analyses did not show effect modification by school-related variables. The odds ratio of having talked to a student counsellor was highest for adolescents with multiple ill parents, compared to no ill parents. Conclusions: Parental illness is a strong predictor of low life satisfaction among adolescents; the impact depends on number of ill parents, whether parental illness is physical or mental, and their level of impairment. Positive school experiences were less frequent in adolescents of ill parents and did not counteract the effect of parental illness on life satisfaction.

## 1. Introduction

The prevalence of children having a parent with a chronic or serious physical illness vary between studies and methods used to estimate the prevalences. Barkmann et al. (2007) estimated that 4.1% of 4–18-year-olds in Germany had a parent with a serious physical illness [1]. An Australian review suggests that up to one in five young people had a parent with a mental illness [2]. When a parent becomes ill, the whole family is affected. Studies have shown associations between parental illness and emotional and behavioral problems among the children, and parental illness may even hamper the psychosocial development of the child [3,4,5,6,7,8]. Moreover, parental illness has been associated with lower grade point average (GPA), higher risk of low educational achievement, and attenuated income in adulthood [9,10,11,12]. These consequences of parental illness may be a result of the role reversal, reduced parental capacity, and lack of parental attention, which has been found to be common consequences and coping-mechanisms in families facing parental illness [13,14,15,16]. 

Adolescents’ satisfaction with life has shown to be affected by many different factors including individual factors and contextual factors [17]. A positive school climate has been associated with academic, behavioral, and socio-emotional outcomes among students [18,19,20] and students perception of school climate has been identified as an important predictor of life satisfaction [21,22]. School climate has been defined as *“the norms, expectations, and beliefs that contribute to creating a psychosocial environment that determines the extent to which people feel physically, emotionally, and socially safe”* [19] and is often studied in relation to four sub-constructs: social connectedness/relationships, school safety, school connectedness, and academic environment [19]. In this study, we focus on the individual’s experiences in school in relation to the community in the classroom and their relationship with teachers. These factors relate to the connectedness and relationship component in the concept of school climate [23].

From a theoretical point, resilience provides a useful framework for understanding why a positive school experience may act as a protective factor, moderating the negative impact of risk factors, for instance parental illness [24]. School may enhance the resilience of adolescents affected by parental illness by offering an opportunity for meaningful activities, a sense of belonging, and a source of respite [24,25,26]. For adolescents facing adversities at home, teachers and student counsellors may be trusted adults who are not part of the family, which has shown to be of great importance for the resilience of children [24,27]. The potential moderating effect of school climate has been investigated in relation to family structure and family poverty. O’Malley et al. (2015) found a stronger effect of a positive school climate on self-reported grade point average among homeless students and students from one-parent homes [28]. Hopson and Lee (2011) found that positive school climate perception attenuated the negative effect of family poverty on problem behaviors among students in middle and high school [29].

In summary, studies have shown that parental illness can have a great impact on everyday life of the children. School climate have been positively associated with academic and social-emotional outcomes among students and studies even suggest a moderating effect for students at risks. However, little is known about the potential of positive school experiences to compensate or moderate the risk of poor well-being among children living with parental illness. In this study we intend to address this gap in the literature by: (1) Examining the relationship between parental illness and life satisfaction and between school-related factors (classroom community, academic support from teachers and trust in teachers) and life satisfaction among adolescents; (2) investigating the possible modifying effect of school-related factors in the association between parental illness and life satisfaction; and (3) Describing the use of student counselling services among adolescents with and without an ill parent.

## 2. Materials and Methods

### 2.1. Participants

The study is based on data from the Danish study Well-being Despite, a cross-sectional survey including a nationwide sample of schools. A random sample of 198 schools were invited to participate, whereof 66 agreed to participate including primary and secondary schools, boarding schools (children in 9th or 10th grade), 10th grade school-centers, vocational schools, and high schools. Within schools, the participation proportion was 66.6% resulting in a total of 10,893 students. The study population in this article was restricted to students attending 7th grade and above (*n* = 10,320) aged 12–26, 97% between 13 and 19 years-old. Students with missing information on institution number (*n* = 27), parental illness (*n* = 206), life satisfaction (*n* = 36), school-related factors (*n* = 338), age (*n* = 100), or gender (*n* = 48) were excluded, resulting in a study sample of 9565 adolescents.

A thorough development process preceded the final questionnaire, including qualitative interviews with the target group, consulting a panel of experts, evaluation of the questionnaire by a children’s panel, and pilot testing in different school settings. Data were collected from September–November 2016. Students completed a web-based questionnaire during school-hours with a teacher present for assistance. Students used approximately 30 min to complete the questionnaire. Prior to the data collection, teachers, parents, and students received written information about the project. This included information about relevant organizations to contact if the students felt a need to talk about any difficult issues. In Denmark, research projects not including human biological material, are not obliged to obtain acceptance by an ethical committee. The study is affirmed at the Data Protection Agency at the University of Southern Denmark (J.nr. 10.755).

### 2.2. Measures

#### 2.2.1. Life Satisfaction (Outcome)

Life satisfaction was measured by an adapted version of the Cantril Ladder [30]. The students were presented with a vertical visual scale (ladder) ranging from 0 to 10, with the following statement: “*How are you doing at present?*” 10 means “*the best possible life for you*” and 0 means “*the worst possible life for you*”. A score of 6 or below was considered low life satisfaction [30]. The Cantril Ladder was used as outcome measure in this study, as we wanted to investigate the individual’s overall evaluation of their satisfaction with life. The adapted version of the Cantril Ladder has been part of the Health Behavior in School Children since 2002. This adapted version of the Cantril Ladder is validated and easy to administrate. Moreover, the Cantril Ladder is a generic measure, which allows comparison between adolescents with and without an ill parent [30,31].

#### 2.2.2. Parental Illness (Exposure)

Students were asked “*Do any of the following persons have a serious illness, chronic illness, or a disability?*” with response options: *mom, dad, stepmom, stepdad, sibling, no,* followed by the question “*What illness/disability does your mom/dad/stepparent have*?” with response options: *migraine or frequent headache, apoplexy, visual impairment, hearing impairment, spinal conditions, cancer, arthritis, sclerosis, mental illness (e.g., depression or anxiety)*, heart disease, kidney disease, other illness/disability (an open-ended question). Moreover, students were asked “*Think about the past month. How often were your parent(s) unable to participate in activities because of his/her illness/disability?*” with response options *seldom or never, less than once a week, once a week, 2–5 times weekly, daily or almost daily, several times a day*. The face validity of questions concerning parental illness was tested in children’s panels and during pilot testing. Parental physical illness was categorized as functionally impairing if the student responded *once a week, 2–5 times weekly, daily* or *almost daily,* or *several times a day*. As initial analyses revealed that students with a mentally ill parent, regardless of impairment, differed significantly from adolescents with a healthy parent in relation to life satisfaction, parental mental illness was not categorized according to impairment. In this study, we included illness of mothers, fathers, and cohabiting stepparents. The students were categorized as follows: (1) No ill parents, (2) One or multiple physically ill parents with no functional impairment, (3) one physically ill parent with functional impairment, (4) one mentally ill or mentally and physically ill parent, (5) multiple ill parents, at least one of them mentally ill or physically ill with functional impairment.

#### 2.2.3. School-Related Factors

Student–teacher relationship was measured by two items: “*I feel that I can trust my teachers?*” and “*I wish the teachers at my school would ask more about how I am doing academically*”. The variables were dichotomized into fully agree/agree vs. neither agree nor disagree/ disagree/ strongly disagree. One indicator of the community in the classroom was included: “*Do you feel as part of the community in your school class*”, response options dichotomized into always/most of the time vs. sometimes/seldom or never/there is no community in my class. The face validity of the items concerning school experience was tested through children’s panels and pilot testing and revised until satisfactory. The question concerning trust in teachers has been developed by HBSC network.

The students’ experiences with student counsellors were captured by the flowing questions: “*Have you talked to a school counsellor at the school about specific challenges you have (e.g., your own illness or family illness)?*” with response options: *Yes, several times; yes, one time; no; there is no student counsellor at my school*. If no: “*Would you like to talk to a student counsellor?*”, response options *Yes; no; don’t know*. If yes: “*I received good help/support from the student counsellor*”, response options *fully agree; agree; neither nor; disagree; strongly disagree*.

#### 2.2.4. Combined Variables

To investigate the joint effect of school-related factors and parental illness we combined information about parental illness and the three indicators of a positive school experience into three new categorical variables with ten categories each. (1) Trust in teachers plus parental illness. (2) Academic support from teachers plus parental illness. (3) Part of the classroom community plus parental illness. The joint reference category allows us to compare each combination of school climate indicator and parental illness according to the same baseline odds.

#### 2.2.5. Socioeconomic and Illness Characteristics

Family occupational social class: Students were asked to state their parents’/stepparents’ occupation. The answers were coded into occupational social class ranging from I (high) to V (low), VI economically inactive parents receiving transfer income, and VII unclassifiable occupation, studying, or missing. These categories were collapsed into high (I and II), medium (III and IV), low (V and VI), and unclassifiable/students/missing (VII) [32]. The highest ranking parental occupational social class was used as an indicator of the adolescents’ family occupational social class.

Students were categorized as having a physical or mental illness based on the question *“Do you have…”* followed by a list of somatic and mental diagnoses and the response options *“other physical illness”, “other mental illness” and “I have no illnesses, diagnosis or disabilities”.*

The school-related variables, socioeconomic variables and questions concerning parental illness were developed for the Well-being Despite Study or originate from the Health Behavior in School-aged Children (HBSC) [33].

### 2.3. Statistical Analyses

Analyses were performed using SAS 9.4 (SAS Institute Inc, Cary, NC, USA). Due to the hierarchical structure of data, we performed multilevel logistic regression analysis. The level 1-units were students and level 2-units were schools. Information about the organization of students in school classes within schools was not available. To investigate the possible modifying effect of school-related factors, we performed multilevel logistic regression analyses using the combined variables. Moreover, multilevel logistic regression analyses were performed including interaction terms between parental illness and the school-related factors. Statistical significance was set to *p* < 0.05. Sensitivity analyses were performed, using a score of 5 or below on the Cantril Ladder as the outcome measure, as this cut-off has also been used in previous studies to represent low life satisfaction [30]. Moreover, sensitivity analyses adjusting for students’ own illnesses were performed, as there is a risk that students’ own illness may have affected the development of parental mental illness and thus be a confounder.

## 3. Results

A total of 1424 students (14.9%) reported parental illness (Table 1). The proportion of students reporting mental illness of a parent(s) was 3.8%. Approximately half of the students reporting no ill parents were female while a larger proportion of the students reporting an ill parent were female. A smaller proportion of the adolescent with an ill parent had high family occupational social class and a larger proportion reported mental/behavioral or physical illness, compared to adolescents who did not have an ill parent (Table 1). A larger proportion of adolescents with ill parents reported poor school experiences and low life satisfaction than adolescent without ill parents.

Most of the adolescents with an ill parent had an ill mother and have had an ill parent(s) for at least four years (data not shown). The most frequently reported physical illnesses for parents with and without functional impairment were spinal conditions (e.g., herniated disc), migraine or frequent headaches, arthritis, and cancer. Information about the specific mental illness was not available.

### 3.1. Association between Parental Illness, School-Related Factors, and Life Satisfaction

The adjusted OR of low life satisfaction was significantly higher among adolescents with ill parent(s), compared to adolescent without, except for adolescents with physically ill parent(s) without functional impairment (Table 2). Changing the cut-point for low life satisfaction from 6 to 5 on the Cantrill Ladder did not alter the results (results not shown in table). Adjusting for own physical and mental illness of the adolescents resulted in slightly smaller effect estimates but did not alter the overall findings (results not shown).

The OR of low life satisfaction was significantly higher for students who did not agree to the statements “I can trust my teachers” (OR 2.13, 95% CI: 1.91–2.37), “I wish the teachers at my school would ask more about how I am doing academically” (OR 1.44, 95% CI: 1.29–1.61), and students who did not feel part of the classroom community most of the time/always (OR 3.91, 95% CI: 3.47–4.41). The between-school variations, Intraclass Correlations, were between 3% and 4% in the associations between parental illness status, school-related factors, and life satisfaction (results not shown).

### 3.2. Joint Effect of School-Related Factors and Parental Illness on Life Satisfaction

**Figure 1:**
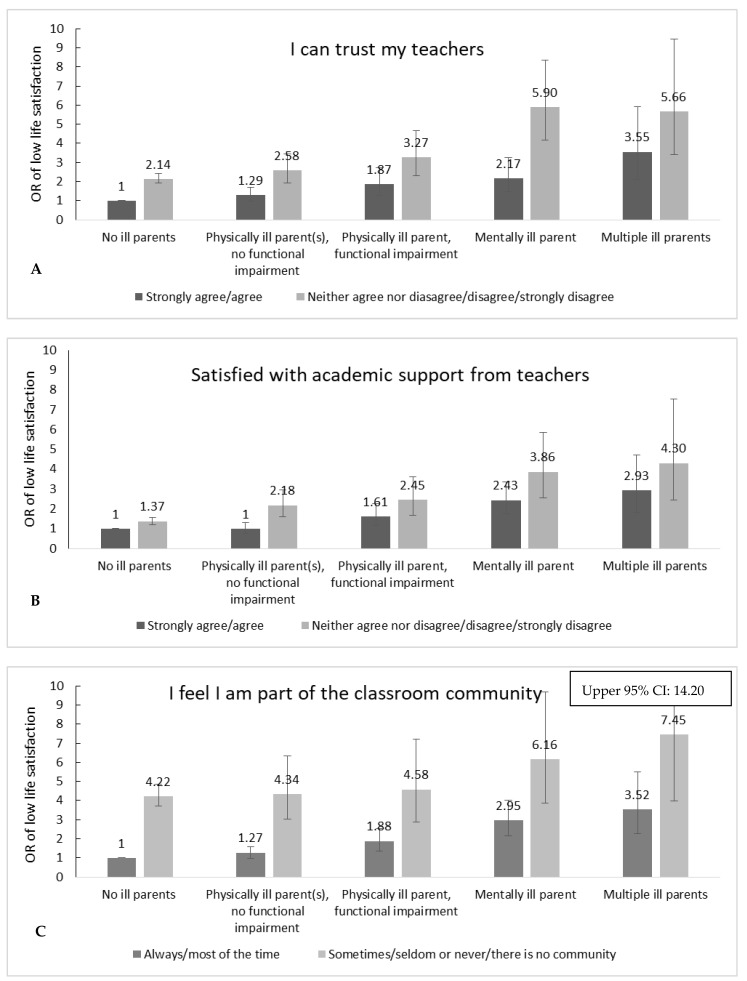
Odds ratios of low life satisfaction by joint effect of parental illness and school-related factors. Adjusted for gender, age, and family occupational social class.

The joint effect of parental illness and trust in teachers (Figure 1A) showed that the highest OR of low life satisfaction was found among adolescents with a mentally ill parent and low trust in teachers (OR 5.90, 95% CI 4.17–8.34) compared to adolescents with no ill parents who trust their teachers. The multiplicative interaction term was not significant (*p* = 0.69). The joint effect of parental illness and academic support from teachers (Figure 1B) resulted in an OR of low life satisfaction of 4.30 (95% CI 2.45–7.54) for adolescents with multiple ill parents who responded neither agree nor disagree, disagree or strongly disagree with the statement “I wish the teachers at my school would ask more about how I am doing academically”. The multiplicative interaction term was not significant (*p* = 0.26). The joint effect of parental illness and not being part of the classroom community (Figure 1C) was highest among adolescents with multiple ill parents (OR 7.44, 95% CI 3.91–14.20). The interaction term was significant (*p* = 0.02). The interaction estimate showed that, on a relative scale, being part of the classroom community was not as strongly associated with life satisfaction among students with an ill parent as for adolescents with no ill parents.

### 3.3. Students’ Experiences with Student Counsellors

The OR of having talked to a student counsellor was highest among adolescents with multiple ill parents compared to adolescents with no ill parents (OR 2.42, 95% CI 1.65–3.55) (Table 3). Between half and two-thirds of the students who had talked to a student counsellor found the counselling helpful. Among students who had not talked to a student counsellor, the vast majority did not express a wish to do so. However, more adolescents with a mentally ill parent or multiple ill parents wished to talk to a student counsellor compared to adolescents with no ill parents.

## 4. Discussion

This study supports the previous findings of an association between positive and supportive relations in school and well-being among adolescents [15,17,30]. This association was found among adolescent across the parental illness categories, suggesting that positive school experiences operate as promotive factors enhancing life satisfaction for all students. Adolescent with an ill parent more frequently reported negative school experiences. However, no support was found for a buffering effect of school-related factors in the association between parental illness and life satisfaction. In the context of resilience theory, perceived as the balance between risk and protective factors acting within a social context, the lack of a buffering effect by school-related factors might be due to the exploration of single indicators of a positive school experience, not using a comprehensive and validated measurement of school climate [34].

Adolescents with ill parents had greater odds of having talked to a student counsellor about troubling issues. Among adolescents who had no experience with student counsellors, students with a mentally ill parent or multiple ill parents more frequently wished to talk to a student counsellor. Moreover, a substantial proportion of the students did not know, if they wished to talk to a student counsellor, indicating a lack of knowledge about what the student counsellor can offer.

The association between parental illness and life satisfaction is in accordance with Pakenham & Bursnall [35]. We studied parental illness regardless of diagnosis and found great differences in life satisfaction depending on whether the parental illness was physical or mental, related impairment of the illness, and number of ill parents. Differences in the odds of low life satisfaction across parental illness status are in line with the findings of Van der Werf et al. [15] and Krattenmacher et al. [36]. Van der Werf et al. [15] found that students living with a mentally ill parent were at greater risk of negative consequences in their daily life than those living with a physically ill parent. Likewise, Krattenmacher et al. [36] found a higher frequency of emotional and behavioral problems among children living in families with parental mental illness, than in families with parental cancer. A greater vulnerability in children of parents with mental disorders may partly be explained by the heritability of mental illness [37]. However, sensitivity analysis revealed that the effect of parental mental illness on life satisfaction remained when adjusting for mental illness of the child. Our results also show that the most frequently reported diagnoses by adolescent with a physically ill parent without functional impairment and a physically ill parent with functional impairment were the same. This underlines the importance of measuring impairment and not only diagnoses, as the same diagnoses may have very different impact on the life satisfaction of the child depending how much the illness impairs the parent. Likewise, Stoeckel and Weissbrod [4] found that greater incapacitation and disruption of everyday activities by the parental illness was negatively correlated with adolescent’s risk of depression, anxiety, and life satisfaction. The variation in the association between parental illness and life satisfaction according to the type of parental illness may be due to variation in the impact of the illness on e.g., parental capacity, parental attention, and potential role reversal, which have been identified in qualitative research as potential consequences of parental illness [13,14,15,16]. It is plausible that the more impaired the ill parent is, the more likely it is that for instance parental capacity is affected, resulting in higher risk of low life satisfaction among the children. The mechanisms underlying these variations in life satisfaction among adolescent with an ill parent should be studied further.

### Strengths and Limitations

To the best of our knowledge, this is the first study to investigate positive school experiences as potential protective factors for adolescents growing up with an ill parent and to explore the use of student counsellors in relation to parental illness. The study population included a large and nationwide sample of students from all types of youth educations. In contrast to many previous studies, adolescents were not recruited through their ill parent or as a result of contact with the healthcare system; in this study parental illness was identified in a large, random sample of adolescents, reducing the risk of selection bias.

Life satisfaction was measured by a single, self-reported, and validated measure, the Cantril Ladder [30]. The measurement of positive school experiences was also based on self-report by the adolescents. A German study of school-aged children found that students’ perception of the climate in the classroom to be a strong predictor of life satisfaction, whereas the overall learning climate in school classes were only weakly associated with life satisfaction [22]. This indicates that the perception by students of school-related factors, as used in this study, are more important to life satisfaction, than objective measures of e.g., school climate.

There are several limitations to consider in this study. Due to the cross-sectional design, causal directions among variables cannot be established. It is unlikely that low life satisfaction among children will cause parental illness, yet low life satisfaction may affect the reflection on and sensitivity to parental illness; the effect of this on adolescents’ answers to parental illnesses is unclear. Moreover, low life satisfaction may lead to a more adverse assessment of school-related factors. The prevalence of parental mental illness in our study is markedly lower than previous estimations [2]. This could be due to the phrasing of the question concerning parental illness, including the words “serious” and “chronic”, which might not be ideal in the assessment of parental mental illness. Moreover, presenting a list of mental illnesses could have reminded students of potential parental mental illness. Nondisclosure of parental illness could also be due unwillingness to report, taboo in relation to mental illness [38], or because the adolescent was unaware of the illness. The notable gender differences in reporting of parental illness, with lower prevalence among male respondents, could be due to differences in the experience of and response to having an ill parent. Studies have shown that female offspring take on more caring activities [11,39,40] and have a higher risk of negative outcomes [41,42,43,44,45] in relation to parental illness, compared to male offspring. Under-reporting of parental illness might have led to misclassification of students with ill parents as having no ill parents.

We included all types of parental illness, regardless of diagnosis, comprising also rare diagnoses. Collapsing a continuum of illness into broad categories does however pose the risk of averaging out significant differential effects. For instance, we were not able to consider if the parental illness was life-threatening or not. Adolescents attending high school were overrepresented in the study population and the study excluded adolescents not attending a youth education. There is a potential risk that we explored a particularly high functioning sample of adolescents, if those who are most negatively affected by parental illness does not attend youth educations or more often attend vocational education programs. This may have introduced selection bias and caused an underestimation of the effect of parental illness on life satisfaction.

## 5. Conclusions

This study demonstrates a strong association between parental illness and life satisfaction. The variation in strength of the associations across the different categories of parental illness (mental, physical, and functionally impairing) suggests that when intervening in families with parental illness, it might not only be the diagnosis that determine whether life satisfaction of the children is at stake, but also the degree of impairment and interruption of everyday life.

Life satisfaction among adolescents with and without an ill parent was positively associated with positive school experiences. School climate indicators did not act as an effect modifier in the association between parental illness and life satisfaction. However, adolescents with ill parents more frequently reported negative school experiences. This underlines the importance of school personal to actively ensure a supportive student–teacher relationship and inclusion in the classroom community when students face parental illness. Student counsellors might be a potentially unrecognized source of support for students experiencing parental illness. Future research should explore the gender differences in reporting of parental illness and the potential supportive role of the school, including teachers and student counsellors.

## Figures and Tables

**Table 1:** Characteristics of the study population by parental illness: socioeconomic, demographic, own illness, school-related factors, and life satisfaction.

	No Ill Parents	Physically Ill Parent(s), No Functional Impairment	Physically Ill Parent, Functionally Impaired	Mentally Ill Parent	Multiple Ill Parents	*p* ^1^
**Total** (% (*N*))	85.1 (8141)	7.2 (688)	3.4 (325)	2.9 (282)	1.4 (129)	
**Age** (mean (SD))	16.9 (1.45)	16.9 (1.40)	17.2 (1.59)	17.0 (1.67)	17.4 (1.61)	<0.0001
**Age range**	12–26	12–25	12–26	13–26	13–25	
**Age 95% quantile**	19	19	19	20	19	
**Gender** (% female)	55.3	63.8	68.6	74.6	81.4	<0.0001
**Family socioeconomic position** (%)						<0.0001
High	47.7	42.0	34.5	34.4	19.4	
Medium	29.9	33.0	28.6	29.8	27.9	
Low	6.5	11.3	19.1	13.1	30.2	
Students/missing/ unclassifiable	15.9	13.7	17.9	22.7	22.5	
**Own physical illness** (%)	30.9	41.7	48.3	43.6	54.3	<0.0001
**Own mental/behavioural illness** (%)	18.1	21.5	22.8	35.8	53.5	<0.0001
I can trust my teachers (% neither agree nor disagree/disagree/strongly disagree	40.0	37.5	45.9	51.1	49.6	<0.0001
**Satisfied with academic support from teachers (% neither agree nor disagree/disagree/strongly disagree)**	32.7	32.3	37.9	35.1	41.9	0.0803
**I feels I am part of the classroom community (% sometimes/seldom or never/there is no community)**	16.1	18.2	24.9	28.0	31.8	<0.0001
**Low life satisfaction** (%)	16.9	20.8	29.2	37.9	47.3	<0.0001

^1^ Chi-square test or ANOVA as appropriate.

**Table 2:** Odds ratio of low life satisfaction by parental illness and school-related factors.

	Crude OR (95% CI)	Adjusted OR ^1^ (95% CI)
**Parental illness**		
No ill parents	1.00 (ref.)	1.00 (ref.)
Physically ill parent(s), no functional impairment	1.27 (1.04–1.54)	1.20 (0.98–1.46)
Physically ill parent, functional impairment	1.98 (1.54–2.53)	1.70 (1.32–2.20)
Mentally ill parent	2.98 (2.32–3.83)	2.60 (1.99–3.31)
Multiple ill parents	3.98 (2.79–5.69)	3.05 (2.12–4.38)
**I can trust my teachers**		
Strongly agree/agree	1.00 (ref.)	1.00 (ref.)
Neither agree nor disagree/disagree/strongly disagree	2.28 (2.05–2.54)	2.13 (1.91–2.37)
**Satisfied with academic support from teachers**		
Neither agree nor disagree/disagree/strongly disagree	1.00 (ref.)	1.00 (ref.)
Strongly agree/agree	1.53 (1.37–1.70)	1.44 (1.29–1.61)
**I feel I am part of the classroom community**		
Always/most of the time	1.00 (ref.)	1.00 (ref.)
Sometimes/ seldom or never/there is no community	4.25 (3.78–4.79)	3.91 (3.47–4.41)

^1^ Adjusted for gender, age, and family occupational social class.

**Table 3:** Student counsellor experience by parental illness, % (*n*) and odds ratio.

	% (*n*)	Adjusted OR ^1^ (95% CI)	Type III Test, *p*
***Talked to student counsellor, N = 9280***			<0.0001
No ill parents	17.1 (1342)	1.00 (ref.)	
Physically ill parent(s), no functional impairment	20.6 (141)	1.26 (1.03–1.54)	
Physically ill parent, functional impairment	28.6 (92)	1.74 (1.34–2.26)	
Mentally ill parent	35.7 (99)	2.52 (1.94–3.29)	
Multiple ill parents	38.3 (49)	2.42 (1.65–3.54)	
**Among students who talked to a student counsellor, *N* = 1725**			
***Strongly agree/agree that it was helpful***			0.31
No ill parents	61.2 (820)	1.00 (ref.)	
Physically ill parent(s), no functional impairment	61.4 (86)	1.03 (0.72–1.48)	
Physically ill parent, functional impairment	51.1 (47)	0.71 (0.46–1.09)	
Mentally ill parent	67.7 (67)	1.36 (0.89–2.12)	
Multiple ill parents	59.2 (29)	1.01 (0.56–1.82)	
**Among students who did not talk to a student counsellor, *N* = 7478**			
***Wish to talk to a student counsellor***			0.0003
No ill parents	8.8 (565)	1.00 (ref.)	
Physically ill parent(s), no functional impairment	6.3 (34)	0.69 (0.48–1.00)	
Physically ill parent, functional impairment	9.7 (22)	1.14 (0.72–1.81)	
Mentally ill parent	11.8 (21)	1.52 (0.94–2.46)	
Multiple ill parents	21.8 (17)	3.04 (1.69–5.46)	
***Do not know if they wish to talk to a student counsellor***			0.32
No ill parents	23.6 (1523)	1.00 (ref.)	
Physically ill parent(s), no functional impairment	22.4 (120)	0.86 (0.69–1.06)	
Physically ill parent, functional impairment	24.3 (55)	0.96 (0.70–1.32)	
Mentally ill parent	30.3 (54)	1.31 (0.93–1.84)	
Multiple ill parents	23.1 (18)	1.00 (0.57–1.75)	

^1^ Adjusted for gender, age, and family occupational social class.

## Data Availability

The datasets used during the current study are available from the corresponding author on reasonable request.
